# Validity of an Instrument to Detect Cheating Confirmed by the Elicited Emotional Reactions

**DOI:** 10.3389/fpsyg.2021.635228

**Published:** 2021-12-20

**Authors:** Paola Hernández-Chávez, Jonatan García-Campos, Saúl Sarabia-López, Daniel Atilano-Barbosa, Alejandra Rosales-Lagarde, María Leticia Bautista-Díaz

**Affiliations:** ^1^Instituto de Ciencias Sociales, Universidad Juárez del Estado de Durango, Durango, Mexico; ^2^Instituto de Investigaciones Filosóficas, Universidad Nacional Autónoma de México, Mexico City, Mexico; ^3^Instituto de Neurobiología, Universidad Nacional Autónoma de México, Mexico City, Mexico; ^4^Cátedras CONACyT, Consejo Nacional de Ciencia y Tecnología, Mexico City, Mexico; ^5^Instituto Nacional de Psiquiatría Ramón de la Fuente Muñiz, Mexico City, Mexico; ^6^Facultad de Estudios Superiores Iztacala, División de Investigación y Posgrado, Universidad Nacional Autónoma de México, Mexico City, Mexico

**Keywords:** emotional reactions, cost-benefit, detect cheating, questionnaire, content validity, construct validity, vignettes

## Abstract

Cheating forms part of a complex emotional and cognitive process. However, although a relatively mundane phenomenon, instruments to evaluate cheating and its effects socially are scarce. This paper presents a five-stage approach aimed at providing validity to an instrument designed to assess cheating — specifically, its detection, and emotional reactions towards it once detected. An instrument was designed after (1) reviewing the relevant literature on cheating, in order to (2) design a bank of stimuli, (3) formulate a Delphi panel to judge the most coherent and pertinent ones, and (4) perform three pilot studies to adjust the final version of the instrument. Results from Stages 1 to 4 show that content validity was achieved for the Instrument for Detecting Cheating and its Emotional Reactions (INDETRAE, in Spanish: *Instrumento para la Detección de Trampa y sus Reacciones Emocionales*). Stimuli were grouped into five categories of 18 different scenarios, for a total of 90 vignettes: meaning, the INDETRAE is a 5-category, vignette-based questionnaire consisting of contrasting social cost-benefit scenarios, where the cheating situation affects an undefined, a first or a third person, and also a neutral category with no cheating. In Stage 5, several chi-squared tests (*p* < 0.0005) revealed significant differences between categories, proving that the instrument can indeed be used to detect cheating and to identify differentiated emotional reactions – for example, anger when there was detriment to a third person as opposed to neutral situations, or glad when there was a case of cheating which benefited the first person. The last stage counts as the first approximation to support construct validity of the INDETRAE. The most important contribution of this work consists in developing an instrument to detect cheating, confirmed by the resulting emotional reactions, which therefore demonstrate its validity.

## Introduction

### The Detection of Cheating

Various influential authors have argued that cheating is a widespread human behavior and they have maintained the classic discourse that human beings can detect cheaters because possessing this skill played a crucial role in the evolution of our species (Cosmides and Tooby, 1992; Ermer et al., 2006; Cosmides et al., 2010). Meanwhile, according to other literature, there is a high tolerance to self-benefiting behaviors, explainable by habituation, suggesting that individuals in corrupt societies are so exposed to these circumstances that they become inured and are no longer able to distinguish them. Of these opposing views, can both be true? Does our species now need a process of adaptation to dishonesty in order to evolve further? (See for example, Garrett et al., 2016; Gächter and Schulz, 2016; Shalvi, 2016).

Characterizations of cheating concur in conceptualizing cheating as a violation of a social norm to obtain a particular benefit; for example, in political corruption, it is defined as the use of the public power to obtain a private–personal benefits (Morris, 1991). Other literature on cheating describes it as the incidence of a subject breaking a social rule and/or receiving a benefit without paying for it (Grèzes et al., 2004; Spence, 2004; Ermer et al., 2006; Ganis et al., 2009; Litoiu et al., 2015).

Thus, a basic definition of cheating can be expressed as follows: the deliberate violation of a social norm to obtain a personal benefit (i.e., any sort of recompense or payoff in terms of resources, assets, dividends, remuneration, social recognition, advantages, rewards, or compensations, etc.); and the cost, literal or figurative, being paid by a secondary, often undefined, agent or group of agents, regardless of whether the society/public is aware of or detects the cheating or not. In some contexts, such as politics and economics, cheating plays a particularly ubiquitous role. It is often linked to ethical rules, social contracts, questions of human nature – whether human beings are intrinsically altruistic or selfish– and the evolution of morality, among many other issues.

Further literature on the topic includes studies on *detecting* cheating (DC) and others on *producing* cheating (PC). DC and PC studies can, moreover, be approached either from the emotional or from the cognitive perspective. That is to say, there is a difference between assessing the emotional reaction of a person when detecting or when producing cheating. DC is not always easy, partly because by default, we take the behavior as trustworthy (Grèzes et al., 2004). Clues for identifying cheating can be found in verbal and non-verbal information; although it has also been claimed that the most successful people in identifying cheating behaviors are those who pay more attention to subtle insights and kinematic patterns, rather than to verbal and non-verbal clues (Ekman and O’Sullivan, 1991; Grèzes et al., 2004). This makes it necessary to further distinguish those DC studies based on verbal clues, from those based on other sorts of behavior.

A well-known DC study by Ermer et al. (2006) postulated the existence of a detecting cheating module in the brain, triggered under social contexts. Ermer et al. asserted that such a module is domain-specific, universal, innate, associated with a specific neural network and, moreover, prone to a pattern of ontogenetic development. A device being domain-specific means it can process information from one and only one particular domain. To a greater degree, evidence for this DC module came from paper-based tests (Cosmides and Tooby, 1992) and some neurological evidence (Ermer et al., 2006). However, that research has since then taken a back seat for different reasons (Sperber and Girotto, 2003; Buller, 2005) and we do not examine it here.

In contrast to the detection of cheating hypothesis by evolutionary psychologists, we believe that detecting and reacting to cheating, and perhaps producing it as well, are *not* the result of a universal, domain-specific module produced by natural selection (Ermer et al., 2006). We conceptualize cheating as compound functions resulting from cognitive and affective components, including attention, memory, deductive inferences, and theory of mind, among other interrelated processes. All these interwoven emotional reactions are triggered differently depending on the associated factors, such as the violated norm and the manner of the transgression itself, the responsible party, the harm caused and the identity of the aggrieved party, the benefit received, and people who profit from it, and so on.

So, if DC is a part of a complex processing system composed of different emotional and cognitive aspects, then the failure to detect or to react to it might be due to specific components or assemblies not working properly, rather than the failure of the whole (this is a reasonable conjecture that could be inferred from related literature Mascaro and Sperber, 2009; Gerrans and Stone, 2008). In the same vein, it has been proposed that not all cognitive capabilities are modular – some of them are adaptations of complex tasks composed of other simpler cognitive and/or affective mechanisms (Hernandez-Chavez and García-Campos, 2020). Accordingly, lacking the ability to detect cheating might be due to failures in the basic components, those that give rise to aggregative ones upon which the DC depends. The following cognitive aspects might be some of the faulty or missing ones: (1) a lack of understanding of the social contract at play, (2) no appreciation if someone violates a social rule, (3) lack of awareness (or interest) if someone is being adversely affected (and who that may be), (4) a mismatch in the corresponding emotional response, or (5) the mechanism simply not being triggered. In short, detecting and reacting to cheating can be affected if at least one of the constitutive elements are displaced or damaged.

There is as yet no literature studying cheating in those cases where the cheated party is different from the subject who detects cheating; nor in cases where, besides cheating, the emotional reaction to this behavior is a significant by-product which can be measured. As such, we perceived the need to design an appropriate tool for assessing these factors.

The instrument we designed, which will be explained in the Results section, is divided into five categories to study cheating, contrasting social cost-benefit situations, and different emotional reactions (the reader can find all the details in the Supplementary Material). We are also aware of the need of incorporating psychometric properties to achieve validity.

The validation process is, by definition, a continuous reformulation and refinement. However, different types of validity can be pursued: content validity, criterion validity, and construct validity. Of the more important ones, content validity focuses on the extent to which a test measures a representative sample of the subject matter or behavior under investigation [American Psychological Association (APA), 2021]. Construct validity assesses the degree to which inferences can legitimately be made from the operationalizations in the study to the theoretical constructs on which those operationalizations were based. According to APA, it is the degree to which a test or instrument is capable of measuring a concept, trait, or other theoretical entity. Specifically, the questionnaire we present aims to evaluate the identification and reaction of the respondents to cheating, i.e., whether respondents can identify, and how they react to cheating. Thus, the challenge in terms of construct validity for an instrument like this would be the extent to which the respondent is actually able to recognize cheating in different scenarios, including those where they receive a personal benefit.

When a questionnaire is designed, it is desirable for the instrument to attain all types of validity. However, this is not always possible. Alternatively, it has been proposed that content validity can be determined from a representative sample, or a hypothetical universe of situations, or indicators, which together constitute the focus of concern for the person interpreting the test (American Educational Research Association (AERA), American Psychological Association (APA), and National Council on Measurement in Education (NCME), 1999). Nonetheless, when working with constructs, making use of this standard becomes complicated. In response to this, Sireci and Faulkner (2014) asserted that this form of validity could also be provided with the following forms of evidences: test quality, domain definition, domain representation, domain relevance, and the appropriateness of the test development process. We consider that the last encompasses the previous ones. Thus, it is relevant to attain consensus in the conceptual definition of our constructs. Care and caution in the design of the items has been pursued, requiring “expert’s agreement” regarding the indicators of the test (items), as well as piloting the previous versions. Altogether, these steps have been satisfied in order to attain content validity.

We are aware that the validation process of an instrument is extensive. Therefore, we assume, in the first place, that content validity can be demonstrated by the following: (1) reviewing the relevant literature on cheating; (2) generating a bank of reliable and proven stimuli; (3) a committee of experts judging the pertinence of the stimuli conforming to the preliminary versions of the instrument; (4) performing pilot studies to adjust the instrument until a final version was reached; and (5) assessing the potential of the instrument to detect cheating using a large sample of university students, as a first approximation to construct validity (Gregory, 2001; Hogan, 2015; Bautista-Díaz et al., 2019).

Our general objective is to provide validity to an instrument to detect cheating and to identify emotional reactions, which comprises a series of vignettes and a questionnaire.

## Materials and Methods

In this section, we describe the five stages of the design and content validity of our instrument to detect cheating and identify emotional reactions (labeled as Stages 1 to 5).

### Stage 1: Reviewing the Existing Literature on Cheating

A careful review of diverse literature was performed to ensure that we were well-versed in different perspectives on cheating (Cosmides and Tooby, 1992, 2015; Sperber and Girotto, 2003; Ermer et al., 2006; Cosmides et al., 2010; Litoiu et al., 2015). It was very important for us to review relevant literature on lying (Ekman and O’Sullivan, 1991; Ekman, 1992; Talwar and Lee, 2008; Ganis et al., 2009; Harada et al., 2009; Shalvi et al., 2011), dishonest behavior (Shalvi et al., 2012, 2015; Garrett et al., 2016; Gächter and Schulz, 2016; Lee et al., 2018), deception (Zuckerman et al., 1981; Couillard and Woodward, 1999; Grèzes et al., 2004; Spence, 2004; Mascaro and Sperber, 2009), corruption (Morris, 1991; Heywood, 2015; Shalvi, 2016), moral judgment (Kohlberg, 1981; Haidt, 2001, 2007; Monin et al., 2007; Reynolds et al., 2010; Leavitt et al., 2016; Lee and Gino, 2018), and altruistic and egoistic behavior (Trivers, 1971; Axelrod, 2006; Gino et al., 2013). This meant that we were able to distinguish cheating from other related behaviors. The most relevant findings, specifically those related to cheating, have been presented in the previous section.

### Stage 2: Generating a Bank of Reliable and Proven Stimuli

We chose a vignette-format instrument, comprising a series of vignettes and a multiple-choice questionnaire about them. Vignettes have generally been defined as written descriptions of real-life situations that may eventually help to predict judgments, decision-making, attitudes, and behaviors, in clinical, legal, anthropological, sociological, or economic research (Alexander and Becker, 1978; Evans et al., 2015). The use of vignettes, especially in the particular circumstances encountered in the detection of cheating, bears the advantage of presenting specific, concrete, everyday situations, instead of directly posing personal and abstract questions, as in the case of interviews or questionnaires. Indeed, one of the earliest studies on vignettes was the study by Walster (1966), which used recorded vignettes to assess the severity of the consequences of an accident, and how an accountability of a hypothetical subject toward a victim could vary. As noticed by Evans et al. (2015), rape and murder are some of the most sensitive assignments for vignettes.

Thus, vignettes present controlled stimuli that confront the participant with concrete hypothetical situations, helping to assess their responses and emotional reactions in the detection of cheating. Vignettes reduce direct confrontation and the possible effects of participants’ feeling observed (the “Hawthorne effect”; Gould, 1996), or evaluated (the “Sentinel effect”; Veloski et al., 2005). Here, a cross-sectional design was chosen, where all participants were presented with a set of multiple vignettes. As Atzmüller and Steiner (2010) stated, a special, systematic structuring of vignettes has to be carefully designed in order to avoid the effects of fatigue; they recommended around 36 vignettes per subject for public opinion surveys.

Extensive seminar sessions were carried out to make sure that the stimuli were consistent. All the stimuli described hypothetical everyday situations. The vignette-format instrument initially contained 21 situations, each subdivided into five categories (as will be explained below, in Section 3), making 105 stimuli in total. Each category (except the neutral baseline) described cheating situations affecting different agents, in particular, contrasting social cost-benefit scenarios: for example, when the subject who detects the cheating *benefits*, or when the cheating affects an undefined, the first or the third person. The categories were also designed to generate different emotional reactions. These were the main goals of our five categories.

### Stage 3: Judging the Pertinence of the Stimuli Conforming to the Preliminary Versions of the Instrument

To ensure that cheating was effectively evaluated, seven expert judges (a Delphi panel: three of them were experts on cheating literature, and the other four were experts on cognitive psychology) discussed the 105 stimuli and their nuances thoroughly until agreement among them was reached (Riano and Palomino, 2015; López-Gómez, 2018).

In the first and second rounds, the goal was to homogenize the baseline scenarios in terms of word count for the five categories (preserving an average of 18–22 words). Nonetheless, the total word count had to be less rigorous after the judges realized that some participants started losing attention: i.e., with the same word count for each vignette, situations were perceived as repetitive or very similar. Maintaining the attention of the participants was very unlikely if apparently homogeneous initial descriptions were repeated. Moreover, participants were less likely to catch the subtle differences between situations if the initial words in every sentence were the same. So, we blatantly dispensed with standardizing the initial descriptions of the five cost-benefit categories, at the risk of losing participant’s cognitive involvement and emotional engagement. This explains the subtle modifications we incorporated across the five different categories for each scenario during the pilot studies. The total duration of the test did not exceed 40 min, to prevent fatigue.

For the first stimulus category, we composed cheating scenarios without a specific injured entity, but rather an undefined one. For example, “You hear about a party where the condition to enter is to wear a costume, but someone attends without a costume and is also enjoying free drinks.” The second category was designed with cheating that was detrimental to the third person, such as, “Your brother is attending a costume party contest, but he lost his costume on the train. You attend the party and realize the winner is using your brother’s costume.” The third category poses cheating scenarios that are detrimental to the first person, i.e., yourself as the participant. For example, “You are attending a costume party contest. Your costume is stolen. The winner is using your costume.” The fourth category presents unadorned examples of a neutral scenario that works as a baseline, simple situations like, “You are invited to a costume party contest.” Lastly, the fifth category presents cheating scenarios with a *benefit* to the first person, yourself: “You are invited to a costume party but when you arrive, you are the only person in costume. A person you like compliments you on your costume.” (See Supplementary Material for further analyses).

To confirm that the participants understood the stimuli perfectly, we asked them to respond to some basic control questions for each one:

“Did you have enough time to read the situation?”

“Is the description of the situation clear enough?”

“Are you able to imagine the situation?”

“Is cheating involved in the situation?”

The possible responses were “Yes” or “No.”

To test our design, and also to confirm whether the participants could discriminate the subtleties of each scenario, we included specific, concrete questions for the participants such as, “Who is the adversely affected party in this situation?” or “Who is the beneficiary in this cheating situation?” The available responses were: “(i) the cheater, (ii) someone else, (iii) the first person/yourself, (iv) undefined, and (v) nobody.”

And crucially, the innovation in our design was that we included an analysis of the emotional component as the correlative factor to achieve meaningful contrasts. We assessed the emotions experienced when a subject successfully identified and reacted to the cheating that took place and, more importantly, the degree of emotional reaction involved in the situation, by asking:

“How does the described situation make you feel?”

The possible options to describe their emotional response were: (i) Glad, (ii) Indifferent, (iii) Annoyed, (iv) Angry.

### Stage 4: Performing Pilot Studies to Adjust the Instrument to the Final Version

#### Participants

Graduate and undergraduate students participated in the pilot studies. Further details are described below (a Summary Table of this stage can be found at the end of the Supplementary Material).

#### Procedure for the Pilot Studies

The three pilot tests were applied at the Centro Lombardo (a public research center located in Mexico City) in a well-illuminated and ventilated meeting room. Each participant (*N* = 16) responded using an electronic device provided by the research center. The stimuli were presented on a slide presentation software.

There were three pilot studies. In the first, the stimuli consisted of a very heterogeneous word count (up to 5 lines). No time restrictions were established to finish reading the situations. Participants took 1 hour and 40 min to complete the study. Given that the time employed to respond to the questionnaire was too long, some stimuli were rewritten.

In the second pilot study, the stimuli became shorter in order to standardize the stimuli extension (word count). Different reading trials were run for 8, 7, and 6 s. It became consensual that each participant was able to finish reading every situation in no longer than 7 s. Therefore, this time frame was set.

Additionally, as the experts noted, it took some time for the participants to familiarize themselves with the computer keys and the test format; some examples were provided at the beginning of the test.

For the third pilot study, the number of stimuli were reduced to 90, eliminating 3 situations that were confusing or where the subjects did not detect that cheating was involved.

Thus, throughout the three pilot studies, the following situations were observed: (a) the number of words and characters for each stimulus had been controlled and homogenized, but this became more relaxed; (b) as the sentences became shorter, their similarity of expression became more differentiated, and a time frame of 7 s was established. Furthermore, an initial description and examples of the test were introduced to promote the familiarity of the subjects with the instrument; and (c) a reduction in the number of stimuli, now only 90, took place, discarding the controversial stimuli that participants did not perceive as involving cheating (see the final version in the Supplementary Material).

### Stage 5: Assessing the Potential of the Instrument to Detect Cheating in a Large Sample of University Students, as an Approximation to the Construct Validity

#### Participants

A non-experimental, prospective, and transversal study was carried out. No independent variables were intentionally manipulated. It consisted of an on purpose and non-retrospective study done at just one moment in time (Zinser, 1992). A non-probabilistic sample of 259 volunteer students from a public university in Durango, Mexico, participated in this stage of the research. The mean age of the total sample was 23.14 years old ± 5.76 (the age range was 18–59 years). The mean age of women in the sample was (*N* = 157, 60.62%) 22.81 years ± 5.46; and the mean age of men in the sample was (*N* = 102, 39.38%) 23.65 years ± 6.21.

#### Materials

As with the pilot studies, the instrument stimuli were projected onto a screen, using slide presentation software, in a computer lab (Supplementary Material). Each participant responded using a personal computer provided by the University. The study took place at two different computer labs at the Juarez University of Durango State, Mexico. The participants were provided with an access code to open the instrument form, which was previously placed in Google Forms. The URL is available upon request. The form asked for the following information: Name, Age, Gender, and Level of Education. Participants were invited to click back and forth until they become sufficiently familiarized with the form, and then to indicate when they were ready to start.

#### Procedure

The research protocol was presented to the University authorities and a Research Committee for approval. Participants were then given an informed consent, as required by the Ethics Committee (Código Ético del Psicólogo, Sociedad Mexicana de Psicología, 2010). Once each subject had voluntarily agreed to participate, she/he was briefly informed about the type of task they would be responding to.

A total of 90 stimuli were presented to the participants on a screen for 7 s each (as described, this was enough time for all participants to finish reading each stimulus), one at a time. They were given in Spanish and applied to a native-speaker population. We took as valid only the instruments that were fully answered. From a total of 300 participants, the unfinished (*N* = 41) instruments – due to voluntary withdrawal, software, or network connection failure – were discarded, representing 13.7% of the participants, and leaving an 86.3% finished response rate.

After informing the participants about the data protection policy, we provided them with some instructions:

*Instructions offered to the participants before opening the form:*

“*In this experiment, we are assessing the human ability to detect and react to cheating, and the cognitive and emotional reactions elicited when those behaviors are witnessed. On the screen, we are going to present a number of written scenarios depicting everyday situations. They are presented for seven seconds each. You will be asked to respond to a series of questions for each one. Please imagine the situation described in each case, no interpretations are necessary.*”

We supplied a couple of definitions of cheating, followed by several examples, until participants verbally reported that the indications were clear enough. We explained:

“*We refer to cheating when encountering any of the following possibilities:*

(i) *An infraction or violation of social and/or moral rules. For example:*

(a) *Using your mobile phone while you drive, which is against the law.*
(b) *Getting on the subway without allowing other passengers to get off first (thus breaking the rule of “let others get off before you get on”).*
(c) *Someone taking your wallet out of your backpack without your permission or knowledge.*

(ii) *A situation where a subject obtains a benefit without paying for it. A benefit can be defined not only in terms of resources, such as money or food, but also in terms of favors, advantages, recognition, etc. For example:*

(a) *Someone has been invited to a potluck gathering where you bring a dish to share. He/she shows up with nothing to share yet eats and pretends he/she brought a dish, regardless.*
(b) *A guest is asked to split the restaurant bill equally, but does not pay the money even though he/she pretends to do so.*

We included some remarks, such as: (1) cheating does not always take place; (2) it is not always clear who the beneficiary or the aggrieved party is; (3) if you have doubts, you can leave spaces blank.”

#### Statistical Analyses

Frequencies, as well as their corresponding percentages, were obtained for each response. For further pairwise comparisons of frequencies, Pearson’s chi-squared tests were carried out, applying Bonferroni corrections. Chi-squared analyses contrast the distributions of each response in order to identify the following: (1) whether cheating was or was not perceived; (2) who the beneficiary of cheating was; and (3) the emotion each category elicited (Tables 1, 2).

**TABLE 1:** Frequencies per answer for confirmed cheating and no cheating mentioned among categories.

	Categories						
Answer	1-CDdU	2-CDdT	3-CDdF	4-noCD	5-CDbF	df = 4 *p(x^2^)*
Confirmed Cheating	4048^a^	3974^a^	4078^a^	254^b^	3145^c^	<0.0005
No cheating mentioned	472^a^	514^a^	448^a^	4262^b^	1312^c^	

*Pearson’s chi-squared tests were performed throughout the categories to compare the number of answers with Bonferroni corrections.*

*1-CDdU, cheating to the detriment of an undefined entity. 2-CDdT, cheating to the detriment of a third person. 3-CDdF, Cheating to the first person’s own detriment. 4-noCD, a no cheating situation, and no detriment. 5-CDbF, cheating to the benefit of the first person. Frequencies with different superscript letters in rows for confirmed cheating or no cheating mentioned between the five cheating categories represent significant differences at p < 0.005 when Bonferroni test for pairwise comparisons was applied. Pearson’s chi-squared test for overall comparisons represent significant differences at p < 0.0005.*

**TABLE 2:** Frequencies per answer for each category, for the adversely affected subject, the benefited subject, and the elicited emotions.

	Categories						
Affected subject	1-CDdU	2-CDdT	3-CDdF	4-noCD	5-CDbF	df = 16 p(x^2^)
Undefined	1227^a^	236^b^	177^c^	258^b^	619^d^	<0.0005
The Cheater	362^a^	142^b^	85^c^	21^d^	885^e^	
Other	1559^a^	3592^b^	274^c^	157^d^	1408^e^	
Nobody	810^a^	203^b^	144^c^	3868^d^	1270^e^	
Yourself	571^a^	312^b^	3850^c^	192^d^	276^b^	
*Benefited subject*						df = 16 *p(x^2^)*
Undefined	229^a^	217^a^	143^b^	442^c^	238^a^	<0.0005
The Cheater	3394^a^	3457^a,b^	3557^b^	122^c^	413^d^	
Other	234^a^	276^a,b^	309^b^	164^c^	167^c^	
Nobody	602^a^	487^b^	431^b,d^	3516^c^	400^d^	
Yourself	63^a,b^	48^a^	85^b^	253^c^	3236^d^	
*Emotion*						df = 12 *p(x^2^)*
Indifference	1416^a^	929^b^	399^c^	3122^d^	1449^a^	<0.0005
Annoyance	1936^a^	1967^a^	1360^b^	329^c^	633^d^	
Anger	1073^a^	1487^b^	2703^c^	129^d^	189^e^	
Glad	79^a^	70^a,b^	47^b^	862^c^	2158^d^	

*Pearson’s chi-squared tests were performed to compare the number of answers for the affected subject, the benefited subject, and the elicited emotion throughout the categories. Probability values were shown as significant after Bonferroni corrections.*

*1-CDdU represents cheating to the detriment of an undefined entity. 2-CDdT represents cheating to the detriment of a third person. 3-CDdF represents cheating to the Cheating to the first person’s own detriment. 4-noCD represents a no cheating situation, and no detriment. 5-CDbF represents cheating to the benefit of the first person.*

*Frequencies with different superscript letters in rows for response options refer to affected subject, the benefited subject, and the elicited emotion among the five cheating categories represent significant differences at p < 0.005 when Bonferroni test for pairwise comparisons was applied. Pearson’s chi-squared test for overall comparisons represent significant differences at p < 0.0005.*

## Results

### Validity of the Instrument

The instrument to detect cheating and the elicited emotional reactions comprises 90 stimuli (scenarios) across 5 different categories (18 stimuli in each category). The categories were as follows:

(1) A situation illustrating cheating detrimental to an undefined entity (such as an institution). We labeled this first category as cheating to the detriment of an undefined entity (1-CDdU).
(2) A situation illustrating cheating detrimental to a defined third person (perhaps not directly known, but somehow familiar to you, for example, a neighbor, a friend, a sibling, etc.). We labeled this second category as cheating to the detriment of a third person (2-CDdT).
(3) A situation including cheating detrimental to the first person himself, meaning that the participant is the adversely affected subject. We labeled this third category as Cheating to the first person’s own detriment (3-CDdF).
(4) A baseline or neutral control situation, which consisted in reporting a simple initial scenario with no additional information. We labeled this fourth category as no cheating and no detriment (4-noCD).
(5) A situation with cheating, but resulting in the benefit of the first person, meaning that the interviewed participant is the beneficiary. We labeled this fifth category as cheating with benefit to the first person (5-CDbF).

In the final version of the instrument, the word count was controlled (an average word count of 19.78, 20.61, 20.89, 19.56, and 21.11, respectively for each category). The character average was also calculated as follows: 108.83, 110.61, 112.83, 105.28, and 116.39 for each category, respectively. The type of content and the homogeneity of the scenarios were standardized as well.

### Detection and Reaction to Cheating Using the Final Version of the Instrument

**FIGURE 1:**
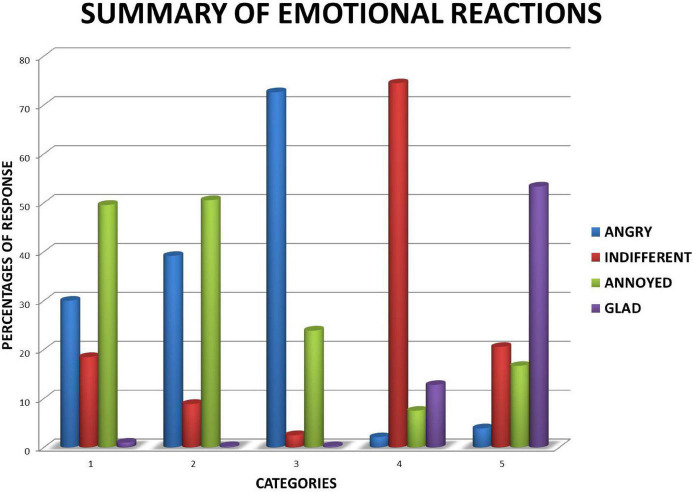
Summary of emotional reactions (indifference, annoyance, anger, and glad).

Employing the described instrument, based on a cost-benefit structure, the elicited emotional reactions for Mexican university students were documented. The results for each category are described below (see Tables 1, 2 and also Figure 1).

#### 1-CDdU: Cheating to the Detriment of an Undefined Entity

For the scenarios in this category, we confirmed that the interviewed subjects correctly recognized the initial condition of cheating, despite the fact that there is not an obviously identifiable “victim.” When we asked each of them to state who the adversely affected party and the beneficiary were, the subjects were able to correctly identify that the cheating was detrimental to an undefined entity. The prototype scenario for this category was something like, “Someone jumps a turnstile at the subway station, i.e., they get into the subway without paying for a ticket.”

Each scenario was followed by our multiple-choice control and assessment questions: “Is cheating involved in the situation?” If so, “Who is the adversely affected party in this situation? (i) the cheater, (ii) someone else, (iii) the first person/yourself, (iv) undefined, (v) and nobody. Who is the beneficiary in this cheating situation?”

We assessed the emotional reactions of the participants with the control question, “How does the described situation make you feel?” The possible options for their emotional reactions were: glad, indifferent, annoyed, and angry (Table 2).

In category 1-CDdU, the most common emotion elicited was “annoyed,” followed by “angry” and “indifferent,” while a very few reported, “glad.” In the posttest interview, we realized that some of those reported “glad” as a result of reading the situation ironically, or even feeling some sort of delight when someone “beats” the established order/system.

#### 2-CDdT: Cheating to the Detriment of a Familiar Third Person

For this category, we again confirmed that the subjects noticed the cheating. As with the previous category, we asked them about the adversely affected party and the beneficiary, whom they were able to identify correctly. The prototype scenario here was something like, “You are attending your friend’s exhibition at a museum. Taking pictures with a flash is not permitted. Someone is taking pictures with a flash and your friend gets in a temper.” Our usual multiple-choice control questions repeated. We assessed our subjects’ emotional reactions with the control question: “How does the described situation make you feel?” and the same four possible reactions (see Table 2).

In category 2-CDdT, like the first one, the most common reaction was “annoyed,” followed by “angry.” Some reported “indifferent,” but almost no one reported “glad” when realizing that the cheating had adversely affected a familiar third person, an acquaintance, or someone they hold in high regard.

#### 3-CDdF: Cheating to the First Person’s Own Detriment

Similarly, for the third category, we confirmed that the interviewees recognized the cheating and the fact that the adversely affected party was the first person, i.e., the participant himself/herself. The prototype scenario here, for instance, was, “In the vehicle testing center, someone pays a bribe to go to the front. Then you are told there are no more tests available today.” We asked our usual multiple-choice control and assessment questions, and again assessed the emotional reactions with the same control questions and the four possible emotions.

This time, in the salient category 3-CDdF, as can be clearly seen in Figure 1, basically only two emotions were elicited: “angry” and “annoyed.” As expected, the participants predominantly reported feeling “angry” when realizing *they* were the directly injured party as a consequence of the cheating.

#### 4-noCD: No Cheating and No Detriment

The scenarios in this baseline control category had no cheating, no adversely affected party, and no beneficiary to report, which the subjects were correctly able to identify when we questioned them. The prototype scenario for this category could be, “Your neighbor invites you to a family party. You imagine that you’ll see all her grandchildren there.” Like the other categories, the same control and assessment multiple-choice questions were used to follow up.

Predictably for the 4-noCD category with no cheating involved, the primary emotional reaction was “indifferent,” with “glad,” and “annoyed” trailing far behind. The respondents rightly realized there was “nothing to see here,” thereby making the argument for the null hypothesis.

#### 5-CDbF: Cheating With Benefit to the First Person

The fifth category presented the converse situation of the third. We confirmed that the subjects could detect the cheating and the affected parties, despite the significant difference: that the participant himself/herself was the *beneficiary* of the cheating. A prototype scenario for this category was, “You are queuing with your friends to enter the bar. One of them is friends with the bouncer and he lets your group in.” As usual, our multiple-choice questions assessed the possible emotional reactions.

Consistent with our expected result, but distinct from all the other categories, the most emphatic response was “glad,” followed at a distance by “indifferent” and “annoyed,” with few interviewees reporting “angry.” So, although they were aware that cheating was taking place, their emotional responses were positive when *they* were the one receiving a benefit.

This is innovative because our hypothesis, supported here by the five-stage study results, bears out a pattern of corruption. Namely, that a *secondary* beneficiary becomes complicit in and *perpetuates* a proself-behavior, i.e., they are aware of the transgression but nevertheless happy to receive a benefit.

As can be appreciated, in the fifth category depicting cheating situations with a *secondary* beneficiary (first person), the resulting emotional reactions were predominantly “glad,” although they may slightly fluctuate depending on who is affected.

The participants were able to identify that cheating occurred in categories, 1-CDdU, 2-CDdT, 3-CDdF, and 5-CDbF. Notably, they did not choose category 4-noCD, and were able to detect that cheating was present regardless of who were adversely affected or whether they were receiving a personal benefit (as in 5-CDbF scenarios).

About 71% of positive identification rate of cheating can be documented among the participants of this study, even if they received a personal benefit, as in 5-CDbF. Accordingly, 29% of the participants failed to report that cheating was taking place in the 5-CDbF scenarios. This is consistent with the diverse emotional reactions elicited solely for 5-CDbF (Figure 1).

### Pearson’s Chi-Squared Tests

#### Cheating

Construct validity is defined as how successfully an instrument represents and measures a theoretical concept (Hogan, 2015; Bautista-Díaz et al., 2019). Once *content* validity of the instrument has been demonstrated, it is necessary to probe complementary forms of validity. As an additional contribution to this study, we investigated a population of university students as the first approach to reaching construct validity. To compare all values contrasting cheating vs. no cheating, a Pearson’s chi-squared test was carried out. Since the INDETRAE discriminates between situations that convey cheating, we moved toward the construct validity.

It was evident that Chi frequencies varied widely using the procedure, for either one option or the other (*x*^2^ = 11094.73, df = 4; *p* < 0.0005). Further Pearson’s chi-squared tests were performed between the pairs of categories with possibly dichotomic answers. There were no differences between the first three categories for the confirmed cheating that was detrimental to an undefined entity, the third or the first persons (1-CDdU, 2-CDdT, and 3-CDdF). Higher frequencies were effectively found when cheating took place in these categories. As expected, the neutral scenario (4-noCD) was less frequently answered. Interestingly, when there was a situation where the first person received a *benefit* from cheating (5-CDbF), compared to the first three categories, cheating was not as high. Also, the frequencies when there was a first person benefiting from cheating (5-CDbF), were significantly higher in comparison to the neutral 4-noCD scenario. First person benefits from cheating doubled and surpassed the first three categories (where no one but the cheater obtained a benefit). More information can be found at the end of the Supplementary Material, for those interested.

#### Detriment

To analyze the global effect of detriment when cheating, another Pearson’s chi-squared test was employed for all the considered frequencies. Results showed a higher significant value (*x*^2^ = 25629.81, *df* = 16; *p* < 0.0005). This was translated into substantial differences among the affected categories. Further chi-squared tests were run for each of the affected categories. It is worth noticing that selecting the “cheater” was the least frequent option chosen, compared to the other possibilities. All the “affected” frequencies varied in the remaining categories. It is important to highlight that, again, for the first category (1-CDdU), the higher frequency corresponded to an undefined entity. Only the third category (3-CDdF) frequencies and the neutral 4-noCD were similar. Frequencies were consistent with the expectations. Higher frequencies corresponded for the option “other,” in the 2-CDdT, 1-CDdU, and even the 5-CDbF categories. The option “nobody” in the 4-noCD and 5-CDbF categories, even when the latter manifested cheating behavior, shows that a detriment was not being perceived at all. Also, the high frequency of “yourself” responses, corresponding to 3-CDdF, was a valid answer. This supports the above-mentioned content validity study (Table 2).

#### Benefit

For this case, the global Pearson’s chi-squared values were highly significant (*x*^2^ = 23430.50, df = 16; *p* < 0.0005). The distribution of frequencies followed a similar scattered pattern in the 1-CDdU, 2-CDdT, and 3-CDdF categories. In this context, the baseline condition of 4-noCD was the most frequently chosen option. In contrast, selecting the cheater option as a response, was more frequent in the 1-CDdU, 2-CDdT, and 3-CDdF. Respectively, 4-noCD was the less frequently chosen option, while the 5-CDbF showed a moderate frequency. The option “other” did not change substantially across the categories. When the option benefit was “yourself,” the category, 5-CDbF got substantially the highest frequency.

#### Elicited Emotional Reaction

A computed chi-squared statistic of 4 rows and 5 columns indicated that the observed results were not obtained by chance (*x*^2^ = 13886.28, df = 12; *p* < 0.0005). Consistently, the option “indifferent” was the most commonly chosen answer in the 4-noCD category. Meanwhile, for the 2-CDdT, frequencies were not so high in contrast to the other ones. The lowest value for the “indifferent” option was obtained for the 3-CDdF category. “Annoyed” was more frequent for the first three categories (1-CDdU, 2-CDdT, and 3-CDdF). Naturally, “anger” and “annoyance” for the 5-CDbF category were not as frequent as for Categories 1, 2 and 3. As was expected, the emotional response “angry” was the most frequent answer for the 3-CDdF, followed by the 2-CDdT, and 1-CDdU. Categories, 4-noCD and 5-CDbF had the lowest frequency for this emotional response. Similarly, for the 5-CDbF category, the most frequently elicited emotional response was that of “glad.” This option was also frequent in the 4-noCD category. The “glad” option was seldom selected in the 1-CDdU, 2-CDdT, and 3-CDdF (Table 2).

From all the above, described in Stages 1 through 5, it can be determined that INDETRAE (in Spanish: Instrumento para la Detección de Trampa y sus Reacciones Emocionales) has been validated, according to the common standardizations (Gregory, 2001; Hogan, 2015; Bautista-Díaz et al., 2019).

## Discussion

As there are currently no instruments of this nature available in the literature to evaluate cheating when the cost-benefit structure changes, we aimed to remedy this by proposing and validating the INDETRAE as described, according to the common standards (American Educational Research Association (AERA), American Psychological Association (APA), and National Council on Measurement in Education (NCME), 1999; Gregory, 2001; Hogan, 2015; Bautista-Díaz et al., 2019). Nonetheless, factor analyses in future investigations could be tested to achieve a stronger construct validity.

The research was conducted in the following five stages: (1) a review of the relevant literature was carried out; (2) a bank of reliable stimuli was generated; (3) a committee of experts judged the pertinence of the stimuli conforming to the preliminary versions of the instrument; (4) pilot studies were performed to adjust the instrument to reach a final 90-stimulus version; and (5) the potential of the instrument was determined to be appropriate to detect cheating in a larger sample of Mexican university students, as an approach to the construct validity.

The INDETRAE demonstrated its capacity for use by university students to detect cheating, as well as to elicit emotional reactions (indifferent, annoyed, angry, and glad), during contrasting situations grouped into five different categories, as we explained during our detailed breakdown of the results for each of these categories. Even in category 5-CDbF, where participants received a personal benefit, 71% were able to detect cheating. This category can thus be extremely conducive to corruption: “You scratch my back and I’ll scratch yours.” This reciprocity, particularly in the percentage of 50-odd respondents who were “glad” to receive their benefit, highlights thorny questions, such as why are dishonest and deceptive behaviors which threaten the public good so insidiously pervasive, and even tolerated by some societies? (Garrett et al., 2016; Shalvi, 2016). Moreover, the 29% of people who do not even *perceive* the cheating subsequently expose why it can also be so hard to legally denounce corruption, as well as why no liability often ends up being the *status quo* (Heywood, 2015), when they fail to recognize cheating for what it is – or indeed cover it up to continue receiving their benefit.

Returning to our INDETRAE results, which confirm that university students *were* able to detect cheating in the four different categories, research shows that during the professional training of the university students, the demand of the students for essay ghostwriters, i.e., hiring someone else to write their essays, has been increasing, with plagiarism becoming a common practice in this population (Rigby et al., 2015). This practice of essentially stealing words and ideas is considered unethical insofar as it harms both the actors and the educational institutions. A recent qualitative study on plagiarism was carried out among Peruvian university students, where it was found that they considered cheating as a common practice from as early as primary education, and perfected all the way up to higher education, since it provides personal and social benefits (such as status). This kind of cheating is *not* perceived as a crime in some populations (Ramos et al., 2019). However, this and other kinds of unethical behavior should be called out and confronted in *any* professional training career. Therefore, the evaluation and communication of these findings becomes imperative in order to sensitize and intervene appropriately among university students.

Furthermore, our INDETRAE results imply that the ability of the university students to detect cheating seems to be general among students. Nonetheless, they do not imply that these capacities are universal or innate, and/or shared by different cultures, as evolutionary psychologists claimed (Cosmides and Tooby, 1992). It would therefore be necessary to look for evidence for when this capacity emerges during childhood (Couillard and Woodward, 1999; Mascaro and Sperber, 2009). A future path for these results could consist in applying this test to subjects of different ages and different political backgrounds; so we can observe the ability to detect cheating during human development.

The use of vignettes for assessing attitudes, beliefs, or judgments, has been proven useful once it allows us to go beyond the dichotomic and unnatural “yes” and “no” answers. Despite the standardization of the latter being much easier to attain (Atzmüller and Steiner, 2010; Evans et al., 2015), content validity is not so obvious. Combinatorial responses and complexity are crucial for content validity, and Delphi panels are a relevant technique for determining such validity (Riano and Palomino, 2015; López-Gómez, 2018). With regard to reliability, given the nature of the multiple-choice responses, it turns out to be complicated to attain an isolated coefficient that allows us to determine reliability. The authors are aware that an “R” software could help estimate the reliability. However, this is beyond the scope of this research, which is limited to providing content validity to the INDETRAE. It is our hope to extrapolate these findings, in the future, in social and political spheres.

## Conclusion

Studying cheating in different cost-benefit situations involves analyzing diverse factors, such as understanding the social contract at stake, realizing someone is infringing a rule within that social contract, and identifying who the beneficiary of that behavior is, and realizing who is being adversely affected.

Of the five stages this study comprised, Stages 1–4 evidence content validity. These included the following: (1) a review of the relevant literature on cheating; (2) the generation of a bank of reliable stimuli; (3) a committee of experts to judge the stimuli in the preliminary versions; (4) pilot studies to improve and develop the instrument into its final version. The last stage (Stage 5), consists of an assessment of the potential of this instrument to detect cheating among a large sample of university students, which counts as an approach toward construct validity. As a result, we can state that there is sufficient evidence to sustain the validity of INDETRAE, but we also recognize that additional evidence has to be incorporated (Gregory, 2001; Hogan, 2015; Bautista-Díaz et al., 2019).

There are, of course, manifold limitations to this research. First, we have not documented whether cheating and deceiving, for example, can be equated. Second, the Robin Hood effect has not been studied – that is, cases where a benefit for a group or a community can be achieved in the name of the common good, even when a rule or norm is violated. Third, if we want to study public/political corruption, then it would be important to evaluate the ability to detect cheating in public office environments, like local, regional and national government, and so on. Another limiting factor is that it is far from clear whether the results we have obtained are consistent with or discredit the polemical cheater detection module postulated by evolutionary psychologists. Additionally, a recognition of an act of cheating by the participants might be inferred from their physical reactions. We presumed that the level of emotional involvement is correlative to the ability of the subject to detect and react to the cheating. If we are right in our prediction, this correlation will show that the closer you are emotionally to a situation, the more acutely you are aware of and feel about cheating. And this should be correspondingly confirmed by the level of intensity in the emotional reaction, which can be assessed by future research. Furthermore, the INDETRAE cheating scenarios could be proposed as task stimuli for neuroimaging research, in order to study the neuroanatomical correlates of cheating stated in this study. Finally, the INDETRAE was applied to intentionally non-probabilistic samples that included a public university student population, and for that reason, it is not possible to generalize our findings. These shortcomings should be taken into account in future investigations.

## Data Availability Statement

The original contributions presented in the study are included in the Supplementary Material, further inquiries can be directed to the corresponding author/s.

## Ethics Statement

The studies involving human participants were reviewed and approved by Instituto de Ciencias Sociales, Universidad Juárez del Estado de Durango. The patients/participants provided their written informed consent to participate in this study.

## Author Contributions

PH-C and JG-C developed the theoretical discussion and designed the experiment. PH-C, JG-C, and SS-L improved the experiment and conducted the interviews and pilots. JG-C and SS-L captured the data. DA-B and SS-L carried out data interpretation and performed the psychometric analyses. PH-C drafted the manuscript and received the support of AR-L and MB-D. AR-L and MB-D reviewed the manuscript format and included the additional psychometric validations. All authors approved the final version of the manuscript for submission.

## Conflict of Interest

The authors declare that the research was conducted in the absence of any commercial or financial relationships that could be construed as a potential conflict of interest.

## Publisher’s Note

All claims expressed in this article are solely those of the authors and do not necessarily represent those of their affiliated organizations, or those of the publisher, the editors and the reviewers. Any product that may be evaluated in this article, or claim that may be made by its manufacturer, is not guaranteed or endorsed by the publisher.
