# Supplementary material for: Validity of an Instrument to Detect Cheating Confirmed by the Elicited Emotional Reactions
Source: Front Psychol. 2021 Dec 20;12:635228. doi: 10.3389/fpsyg.2021.635228 (PMC8720870; doi:10.3389/fpsyg.2021.635228)
Supplement: Supplementary file 1 [file Data_Sheet_1.pdf]

Supplementary Material

1. INDETRAE stimuli and responses per cheating category

As a result of Stages 1 to 4, 90 stimuli (scenarios) were grouped into 18 stimuli each for five different categories that involve cheating. These are: 1) cheating to the detriment of an undefined entity (**1-CDdU**); 2) cheating to the detriment of a defined third person (**2-CDdT**); 3) cheating to the detriment of the first person, meaning *you* are the aggrieved subject (**3-CDdF**); 4) neutral baseline condition (**4-noCD**); and 5) cheating to the *benefit* of the first person, meaning you are the beneficiary in that situation (**5-CDbF**).

For every stimulus/scenario, the participants were asked the following questions:

> "Is there cheating involved in the situation?" The possible responses were "Yes" or "No".
>
> "Who is the adversely affected party in this situation?" The possible responses were: "i) the cheater, ii) someone else, iii) the first person/yourself, iv) undefined, v) nobody.
>
> "Who is the beneficiary in this cheating situation?" The possible responses were: "i) the cheater, ii) someone else, iii) the first person/yourself, iv) undefined, v) nobody.
>
> "How does the described situation make you feel?" The possible options to describe their emotional response were: Glad / Indifferent / Annoyed / Angry.

In what follows, we reproduce in English the Instrument's 90 stimuli (INDETRAE) to detect cheating, from the original ones in Spanish. The initial number corresponds to the order in which they were presented in the vignettes. For each category there are tables that represent the participant responses in percentages related to: cheating detection, the adversely affected, the beneficiary and the emotional reactions involved in each scenario.

1-CDdU. Situations illustrating cheating to the detriment of an undefined entity (such as an institution).

2. You go to the museum to see an exhibition. Taking photos with flash is not allowed. You see someone taking a photo with the flash.
[Vas al museo a ver una exposición. Está prohibido tomar fotos con flash. Ves que alguien toma una foto con flash].
3. A famous soccer player says that he always faked his drug tests to be part of one of the best teams in the country.
[Un famoso futbolista declara que siempre falsificó sus pruebas antidoping para pertenecer a uno de los mejores equipos del país].
9. Your neighbor invites you to her party. Her grandson is sneakily charging the guests $5.00 to use the bathroom.

[Tu vecina te invita a su fiesta. Su nieto está cobrando indebidamente $5.00 a los invitados por usar el baño].
12. The parking lot is full and you see someone park in a place that is not a parking space.
[El estacionamiento está muy lleno, observas a alguien estacionarse en un lugar que no es cajón de estacionamiento].
20. You are in a university entrance exam, and you see that the student in front has a cheat sheet.
[Estás en el examen de ingreso a la universidad, observas que el compañero de enfrente tira un acordeón].
21. You are on the metrobus and you see a violent-looking middle-aged man vandalizing the seats.
[Vas en el Metrobús y observas a un sujeto de edad mediana y aspecto violento que vandaliza los asientos].
32. You are driving home. Suddenly you notice that the driver next to you takes a prohibited left turn.
[Regresas a casa en auto. De repente observas que el conductor de al lado se da una vuelta prohibida].
35. You are queuing at a bar. You notice that an underage guy enters to enjoy happy hour.
[Esperas en la fila de un bar. Observas que una menor de edad entra a disfrutar de la barra libre].
37. Someone is an expert playing the "shell game". It's well known that this person always cheats and wins when he plays.
[Un sujeto es experto en jugar "dónde quedó la bolita". Es bien sabido que ese sujeto siempre roba cuando juega].
48. You are in the subway car. You see a person graffitiing his name on the window to impress his girlfriend.
[Vas en un vagón del metro. Ves que una persona está grafiteando su nombre en el cristal para impresionar a su novia].
53. You hear a checkout girl saying to her colleague that if money is left over, she never gives it back.
[Escuchas a una cajera comentar con su compañera que siempre que le ha quedado dinero de más no lo devuelve].
55. You find out in the media that a presidential candidate declares that he has stolen money—but only a little bit.
[Te enteras a través de los medios de comunicación que un candidato presidencial declara que ha robado pero poquito].
63. You are driving home. The traffic signal turns red so you stop. A person does not brake and runs the red light.
[Regresas a casa en auto. Te toca el alto y te detienes. Una persona no frena y se pasa el alto].
70. You get to the turnstiles to enter the subway. Suddenly you see someone go under the turnstile.

[Llegas a los torniquetes para entrar al metro. De repente ves cómo alguien se pasa por debajo del torniquete].
82. In the swimming pool, the “take a shower before swimming” notice is clearly visible. You see someone enter the pool without showering.
[En el deportivo, es visible el anuncio de que debes ducharte antes de nadar. Observas que alguien entra a nadar sin ducharse].
83. You're in a very crowded subway car and you feel a pervert touching your butt.
[Vas en un vagón del metro muy lleno y sientes que un sujeto pervertido te está tocando el trasero].
87. You are in line to test your car emissions. The person in front of you pays a bribe for their car to pass the test.
[Estás formado en la fila para verificar tu auto. La persona de adelante da mordida para pasar su verificación].
89. You are in a museum. You see a person crossing the red line on the floor to get closer to a painting.
[Estás en un museo. Ves que una persona cruza la línea roja en el piso para acercarse a una pintura].

1-CDdU RESPONSES - PERCENTAGES (%)												
STIMULI	CHEATING DETECTION		ADVERSELY AFFECTED					BENEFICIARY				
	YES	NO	CH	SE	YOU	UND	NOB	CH	SE	YOU	UND	NOB
**2**	87.1	12.9	9.8	22.0	2.8	43.5	22.0	62.8	3.2	1.2	8.1	24.7
**3**	96.5	3.5	29.7	41.4	0.0	21.9	7.0	79.3	2.3	0.4	5.9	12.1
**9**	74.8	25.2	3.9	35.7	35.7	10.9	14.0	79.3	11.7	0.4	2.7	5.9
**12**	93.4	6.6	5.1	40.5	5.8	30.4	18.3	83.3	7.4	0.4	3.1	5.8
**20**	95.3	4.7	19.5	16.7	9.7	26.8	27.2	72.3	2.3	4.7	4.3	16.4
**21**	69.9	30.1	1.6	49.2	7.4	32.4	9.4	35.0	1.6	0.4	11.3	51.8
**32**	94.1	5.9	5.8	15.6	13.2	34.6	30.7	84.4	2.7	0.0	2.0	10.9
**35**	94.1	5.9	28.2	24.7	3.9	20.4	22.7	62.0	16.9	2.0	4.7	14.5
**37**	77.6	22.4	2.4	44.8	20.2	15.3	17.3	77.4	3.2	0.8	4.4	14.1
**48**	77.3	22.7	1.2	44.1	0.8	36.3	17.6	52.5	13.7	0.8	8.6	24.3
**53**	94.3	5.7	2.0	69.4	2.0	21.8	4.8	87.4	5.7	1.2	4.0	1.6
**55**	95.3	4.7	6.3	33.9	29.1	28.7	2.0	87.0	2.0	1.6	4.3	5.1
**63**	94.7	5.3	12.0	21.3	6.4	24.5	35.7	75.0	3.6	1.6	8.5	11.3
**70**	94.8	5.2	2.8	30.0	16.0	25.6	25.6	88.2	2.0	1.2	2.4	6.1
**82**	94.7	5.3	3.3	27.2	31.3	24.8	13.4	81.0	2.8	2.0	3.2	10.9
**86**	94.5	5.5	4.4	49.6	4.0	33.3	8.7	84.2	4.0	2.0	4.3	5.5
**87**	96.6	3.4	3.4	20.2	37.0	23.9	15.5	86.6	3.8	3.4	2.9	3.4
**89**	87.8	12.2	1.6	32.9	2.8	32.5	30.1	75.2	3.7	1.2	6.1	13.8
CH = Cheater; SE = Someone Else; YOU = First Person/Yourself; UND = Undefined; NOB = Nobody												

**1-CDdU** ELICITED EMOTIONAL REACTIONS				
STIMULI	GLAD	INDIFFERENT	ANNOYED	ANGRY
**2**	1.2	42.7	46.0	10.1
**3**	1.2	35.4	39.4	24.0
**9**	5.5	34.5	45.5	14.5
**12**	0.4	32.3	50.6	16.7
**20**	5.5	39.6	34.1	20.8
**21**	1.2	17.3	42.5	39.0
**32**	0.4	39.2	43.9	16.5
**35**	4.3	47.2	39.4	9.1
**37**	0.4	29.4	46.5	23.7
**48**	2.7	25.0	42.2	30.1
**53**	1.2	27.6	45.9	25.2
**55**	1.6	9.5	30.2	58.7
**63**	1.6	37.9	42.7	17.7
**70**	0.8	32.8	50.2	16.2
**82**	0.4	23.9	53.4	22.3
**86**	1.2	18.1	41.0	39.8
**87**	1.3	20.8	44.1	33.9
**89**	0.4	52.0	36.6	11.0

2-CDdT. Situations involving cheating with detriment to a defined third party (perhaps not directly known to you, but familiar by reference. For example, a neighbor, a friend, a sibling etc.).

1. Someone on your soccer team takes a performance-enhancing drug to get selected. They find out about the doping, and wrongly blame your friend.
[En tu equipo de fútbol alguien se dopa para ser seleccionado, se enteran de un dopaje y culpan erróneamente a tu amigo].
6. To make people vote for him, a presidential candidate declares that even though it's illegal, he will ban all Jehovah's Witnesses.
[Para que voten por él, un candidato a la presidencia declara que aunque sea ilegal expulsará a todos los Testigos de Jehová].
10. At the museum, someone crosses the red line to look at their favorite exhibit. The alarm goes off, and someone else is reprimanded.
[En el museo, alguien cruza la línea roja para observar su obra favorita, suena la alarma y regañan a otra persona].
17. Everyone should take a shower before swimming. Your friend is swimming, and a group of people enter the pool without showering.
[Todos deben ducharse antes de nadar. Tu amigo está nadando y un grupo entra a la alberca sin ducharse].
22. In the subway, someone goes under the turnstile and the stationmaster tells the guard off.
[En el metro, un sujeto se pasa por debajo del torniquete y el Jefe de Estación regaña al vigilante].
24. In the library, which should be in silence, your brother is distracted by the person next to him who is listening to music to pass the time.
[En la biblioteca, donde debe guardarse silencio, tu hermano se distrae porque la persona junto a él escucha música para no aburrirse].
28. A checkout girl gives the wrong change to a customer on purpose, and she keeps the rest.
[A propósito una cajera le da mal el cambio a un cliente y ella se queda con ese dinero].
39. You are in a very crowded subway car. You notice that a stalker is touching a teenage girl's butt.
[Vas en un vagón muy lleno del metro. Observas que un acosador está tocando el trasero de una adolescente].
40. In a friend's exhibition, taking photos with flash is not allowed. Someone does it and your friend get very angry.
[En la exposición de un amigo, está prohibido tomar fotos con flash, alguien lo hace y tu amigo se enoja mucho].
41. On the metrobus, a non-disabled person is in a disabled seat. They do not offer their seat when a person on crutches arrives.

[En el Metrobús una persona no-discapacitada ocupa el lugar de discapacitados, llega una persona en muletas y no le cede el lugar].
44. Someone makes a prohibited left turn so they can avoid the red light. They cut in front of the car next to you.
[Alguien se da una vuelta prohibida para que no le toque el alto y bloquea al coche que está junto a ti].
47. Someone playing the “shell game” makes a bet with a child. They trick the child and win her money.
[Un sujeto apuesta con un niño para adivinar "dónde quedó la bolita". Le hace trampa y le gana todo su dinero].
61. A student is copying in the university entrance exam. The supervisor is sanctioned for not noticing.
[Un estudiante copia para pasar el examen de ingreso a la universidad. El responsable es sancionado por no darse cuenta].
64. A car driven by a non-disabled person beats a disabled driver to a parking space.
[Un auto manejado por una persona no-discapacitada le gana el lugar a una persona que sí es discapacitada].
65. While your friend is waiting to enter the bar, there are people cutting in line to make it to happy hour.
[Mientras tu amigo espera para entrar al bar, hay gente que se está metiendo en la fila para alcanzar la hora feliz].
72. In the vehicle testing center, someone pays a bribe to go directly to the front. The car that was at the front now has to wait longer.
[En el verificentro, un hombre da mordida para pasar sin hacer fila, el siguiente tendrá que esperar más].
76. At a family party, your friend goes to the bathroom and the host’s grandson charges her $5.00 to use it.
[En una fiesta familiar, tu amiga va al baño y el nieto de la anfitriona le cobra $5.00 por usar el baño].
90. You are driving home. A person runs the red light and goes past but causes two cars to crash.
[Regresas a casa en auto. Una persona se pasa el alto, no le pasa nada, pero provoca que choquen dos autos].

2-CDdT RESPONSES - PERCENTAGES (%)												
STIMULI	CHEATING DETECTION		ADVERSELY AFFECTED					BENEFICIARY				
	YES	NO	CH	SE	YOU	UND	NOB	CH	SE	YOU	UND	NOB
**1**	76.5	23.5	12.6	64.2	2.8	10.2	10.2	60.9	7.4	0.5	10.7	20.5
**6**	77.8	22.2	2.7	76.2	2.7	14.5	3.9	58.6	11.3	2.7	14.5	12.9
**10**	83.7	16.3	3.6	92.8	1.6	1.2	0.8	76.2	4.4	1.6	5.2	12.7
**17**	88.2	11.8	2.3	53.1	13.7	16.0	14.8	66.0	7.4	0.8	7.4	18.4
**22**	92.9	7.1	4.0	84.5	1.2	6.0	4.4	87.6	1.6	0.4	2.4	8.0
**24**	66.0	34.0	1.2	72.7	11.9	4.3	9.9	55.7	10.7	1.2	4.7	27.7
**28**	96.9	3.1	2.4	91.4	4.3	1.2	0.8	89.0	7.8	2.4	0.8	0.0
**39**	83.3	16.7	0.8	95.3	0.4	1.2	2.3	82.0	3.9	0.0	2.0	12.1
**40**	88.7	11.3	0.8	87.9	3.5	3.9	3.9	70.7	8.2	1.6	3.9	15.6
**41**	95.3	4.7	1.2	94.9	0.4	0.8	2.7	90.6	5.9	0.4	0.8	2.3
**44**	91.9	8.1	2.0	61.1	27.0	4.9	4.9	74.3	7.3	0.8	6.1	11.4
**47**	98.4	1.6	3.1	93.8	1.9	0.8	0.4	92.2	4.7	1.2	0.0	2.0
**61**	90.5	9.5	17.8	67.8	5.0	4.5	5.0	67.5	6.2	1.2	10.3	14.8
**64**	89.6	10.4	0.8	89.5	0.8	3.2	5.6	84.3	4.8	0.4	4.0	6.4
**65**	95.2	4.8	0.8	64.1	23.8	6.5	4.8	85.1	5.2	0.4	4.8	4.4
**72**	96.7	3.3	0.4	85.7	7.8	4.1	2.0	90.5	1.2	2.5	3.3	2.5
**76**	86.7	13.3	1.6	73.7	15.8	5.3	3.6	85.4	9.3	0.4	2.8	2.0
**90**	94.7	5.3	0.8	89.1	1.2	6.9	2.0	68.7	3.2	0.8	4.4	22.9
CH = Cheater; SE = Someone Else; YOU = First Person/Yourself; UND = Undefined; NOB = Nobody												

**2-CDdT** ELICITED EMOTIONAL REACTIONS				
STIMULI	GLAD	INDIFFERENT	ANNOYED	ANGRY
**1**	0.0	37.4	48.1	14.5
**6**	4.3	30.6	42.0	23.1
**10**	3.6	23.9	50.6	21.9
**17**	1.2	36.5	49.4	12.9
**22**	1.2	23.8	47.2	27.8
**24**	1.2	30.2	55.6	12.9
**28**	0.4	12.3	42.5	44.8
**39**	0.0	5.5	22.7	71.8
**40**	0.4	21.4	50.8	27.4
**41**	2.0	4.7	24.0	69.3
**44**	0.8	22.9	52.5	23.8
**47**	2.7	16.0	41.8	39.5
**61**	8.4	26.5	35.7	29.4
**64**	0.4	18.1	40.7	40.7
**65**	0.4	15.3	56.0	28.2
**72**	0.8	17.3	57.6	24.3
**76**	0.4	25.0	49.2	25.4
**90**	0.0	10.9	30.2	58.9

3-CDdF. Situations containing cheating to the first person's own detriment, meaning that the participant is the *aggrieved* party.

5. In the vehicle testing center, someone pays a bribe to go to the front. Then you are told there are no more tests available today.
[En el verificentro, alguien da mordida para no hacer fila, y a ti te dicen que ya no hay fichas para hoy].
14. You are waiting in line to enter the bar. Someone pays a bribe to go in and take advantage of happy hour, while you have to keep waiting.
[Esperas para entrar al bar, alguien soborna para no hacer fila y aprovechar la hora feliz, mientras tú tienes que seguir esperando].
16. Someone makes a bet with you, playing the "shell game". You bet, then you notice that they are tricking you to win your money.
[Un sujeto apuesta contigo "dónde quedó la bolita". Apuestas y te percatas de que te está haciendo trampa para quitarte tu dinero].
30. You are at a family party. You want to go to the bathroom and the host's grandson secretly charges you to use it.
[Estás en una fiesta familiar. Quieres entrar al baño y en secreto el nieto de la anfitriona te cobra para usarlo].
31. At the museum, someone crosses the red line to get closer to their favorite painting. The alarm goes off and you are reprimanded.
[En el museo, alguien cruza la línea roja para acercarse a su pintura favorita, suena la alarma y te regañan a ti].
36. The car in front of you brakes to answer the cell phone; you brake, but the car behind you doesn't and crashes into you.
[El auto delante de ti frena para contestar su celular, frenas, pero el carro detrás de ti no y te choca].
38. You are swimming in the pool. Someone in your lane is peeing in the pool to avoid going to the washroom because it's cold.
[Estás en la alberca nadando. Alguien en tu carril está orinando en la alberca para no salirse al baño porque hace frío].
45. During an exam, the student next to you ditches his cheat sheet. They disqualify your exam because they think it is yours.
[Durante un examen, el alumno junto a ti tira un acordeón y te quitan el examen porque piensan que es tuyo].
49. In an exhibition of your work, taking photos with flash is not allowed. Someone takes a selfie with the flash, with your artwork in the background.
[En la exposición de tu obra está prohibido tomar fotos con flash. Alguien toma una selfie con flash con tu obra de fondo].

51. In the subway, the person in front of you jumps the turnstile to avoid paying. The officer gets confused and arrests you.
[En el metro, la persona delante de ti se brinca el torniquete para no pagar. El policía se confunde y te detiene].
52. In the library, where you should be quiet, someone sitting next to you is talking to amuse themselves.
[En la biblioteca, donde se debe guardar silencio, sentado junto a ti hay alguien que está hablando para distraerse].
57. You go to the store and realize that the checkout girl is giving you the wrong change, pretending she has made a mistake.
[Vas a la tienda y te percatas de que la cajera te está dando mal el cambio fingiendo que se equivocó].
60. You are driving home, when someone makes a prohibited left turn and cuts in front of you.
[Regresas a casa en auto. En ese momento alguien se da una vuelta prohibida y te bloquea el paso].
73. Someone on your soccer team dopes up to get selected. They find out about the doping, and wrongly blame you.
[En tu equipo de fútbol alguien se dopa para ser seleccionado, se enteran de un dopaje y te culpan erróneamente].
75. You find out that an elected candidate will cancel the scholarship you've been granted, so he can receive a better salary.
[Te enteras de que el candidato electo eliminará la beca que tú tienes con el fin de recibir un mejor salario].
79. A car that does not have disabled plates is parked in the disabled space that you need, since you are disabled.
[Un auto de no-discapacitados se estaciona en el lugar para discapacitados que tú necesitas, dado que tienes una discapacidad].
83. You're in a very crowded subway car and you feel a pervert touching your butt.
[Vas en un vagón del metro muy lleno y sientes que un sujeto pervertido te está tocando el trasero].
84. On the metrobus, someone who is not disabled is sitting in a disabled seat. You have crutches but they do not offer you their seat.
[En el Metrobús, alguien no-discapacitado ocupa el lugar de discapacitados, tú tienes muletas y no te cede el asiento].

3-CDdF RESPONSES - PERCENTAGES (%)												
STIMULI	CHEATING DETECTION		ADVERSELY AFFECTED					BENEFICIARY				
	YES	NO	CH	SE	YOU	UND	NOB	CH	SE	YOU	UND	NOB
**5**	96.5	3.5	5.1	14.0	76.3	2.7	1.9	84.4	6.6	3.5	1.9	3.5
**14**	96.5	3.5	1.9	9.7	74.4	8.1	5.8	89.1	5.1	2.7	1.2	1.9
**16**	96.1	3.9	2.3	1.9	91.5	0.8	3.5	93.4	0.4	1.9	0.8	3.5
**30**	86.0	14.0	0.8	6.2	84.0	3.9	5.1	85.2	9.4	0.4	2.0	3.1
**31**	89.8	10.2	0.8	3.5	94.1	0.4	1.2	78.6	9.7	0.4	1.2	10.1
**36**	70.7	29.3	2.4	5.6	87.3	3.6	1.2	43.7	8.7	1.6	5.6	40.5
**38**	87.9	12.1	1.9	8.9	79.0	8.2	1.9	75.4	5.5	0.8	1.2	17.2
**45**	96.9	3.1	0.8	2.7	95.7	0.4	0.4	82.4	6.3	0.8	3.9	6.6
**49**	87.6	12.4	0.4	8.3	71.4	10.7	9.1	74.9	10.0	1.2	4.8	9.2
**51**	96.0	4.0	1.2	2.0	92.7	2.0	2.0	87.3	6.9	0.0	1.6	4.1
**52**	77.6	22.4	4.5	5.3	75.2	8.5	6.5	54.5	10.2	1.2	6.5	27.6
**57**	95.2	4.8	5.2	10.8	75.5	4.4	4.0	71.5	2.8	7.6	4.8	13.3
**60**	91.5	8.5	1.6	1.2	90.7	3.3	3.3	84.4	4.5	1.2	3.7	6.2
**73**	95.1	4.9	2.0	6.0	87.6	2.0	2.4	79.7	7.2	2.0	4.4	6.8
**75**	85.9	14.1	1.2	6.2	86.0	4.5	2.1	75.6	12.2	4.1	4.9	3.3
**79**	94.8	5.2	0.0	6.8	87.6	3.6	2.0	85.1	6.0	2.0	3.6	3.2
**83**	84.3	15.7	1.6	3.2	91.7	1.2	2.4	81.9	6.3	1.6	2.8	7.5
**84**	93.0	7.0	0.0	6.2	89.3	2.1	2.5	87.2	5.3	0.8	2.5	4.1
CH = Cheater; SE = Someone Else; YOU = First Person/Yourself; UND = Undefined; NOB = Nobody												

**3-CDdF** ELICITED EMOTIONAL REACTIONS				
STIMULI	GLAD	INDIFFERENT	ANNOYED	ANGRY
**5**	0.0	6.6	32.4	60.9
**14**	2.3	8.6	40.2	48.8
**16**	1.2	5.1	33.9	59.9
**30**	0.0	19.6	54.1	26.3
**31**	0.4	4.7	31.3	63.7
**36**	1.6	5.2	21.5	71.7
**38**	0.4	3.5	21.4	74.7
**45**	0.4	2.3	14.5	82.8
**49**	0.8	18.5	41.0	39.8
**51**	1.2	5.7	16.4	76.6
**52**	0.4	24.6	48.0	27.0
**57**	2.0	11.8	41.9	44.3
**60**	1.2	8.3	39.3	51.2
**73**	0.8	6.8	22.5	69.9
**75**	2.0	8.9	24.0	65.0
**79**	0.8	9.2	21.7	68.3
**83**	1.6	3.5	14.6	80.3
**84**	1.7	7.0	24.8	66.5

4-noCD. A neutral baseline control situation, which reports a simple scenario with no additional information.

8. You go to the store and see that there are a lot of people and checkouts open, because it is the end of season.
[Vas a la tienda y observas que hay mucha gente y muchas cajeras porque es fin de temporada].
13. You are in a university entrance exam. You notice that most of the students are as nervous as you are.
[Estás en el examen de ingreso a la universidad. Observas que la mayoría de los estudiantes están más nerviosos que tú].
15. You are driving home and there is a lot of traffic on the streets. The light turns red so you stop.
[Regresas a casa en auto y hay mucho tráfico en las calles. Te toca el alto y te detienes].
19. You go to a museum where taking photos with flash is forbidden. The halls are packed.
[Vas a un museo donde está prohibido tomar fotos con flash y hay mucha gente en las salas].
23. Your neighbor invites you to a family party. You imagine that you'll see all her grandchildren there.
[Tu vecina te invita a una fiesta familiar. Te imaginas que te encontrarás a todos sus nietos ahí].
26. It's Sunday afternoon and you are training with your soccer team for an important match against other teams.
[Es domingo por la tarde y estás con tu equipo de fútbol entrenando para un partido importante contra otros equipos].
27. You are swimming in the pool. The notice that everyone should take a shower before swimming is clearly visible.
[Estás en la alberca nadando. Es visible el anuncio que indica que todos deben ducharte antes de nadar].
29. You are watching the candidate debate and you find out in the media that many other people are watching the debate as well.
[Estás viendo el debate de candidatos, te enteras por los medios de que muchas personas también lo están viendo].
33. You go to the supermarket. You notice that the parking lot is very busy and that many cars are the same color as yours.
[Vas al supermercado. Observas que el estacionamiento está muy lleno y que hay muchos autos del mismo color que el tuyo].
42. You go to the turnstiles to enter the subway and you have to queue to go in because there are a lot of people.
[Llegas a los torniquetes para entrar al metro y tienes que hacer fila para entrar porque hay mucha gente].

50. You are on the metrobus and it arrives at the stop. Several people get on, they fill all the seats, and some remain standing.
[Vas en el Metrobús y llega a la estación. Entran varias personas, se ocupan todos los lugares y algunas quedan de pie].
54. A lot of people are waiting in line at the vehicle testing center, despite the fact it's not the end of the month yet.
[En el verificentro hay muchas personas haciendo fila, a pesar de que todavía no es fin de mes].
59. You go to a museum. There are a lot of people in the rooms and you see there are red lines to avoid approaching the artwork too closely.
[Vas a un museo. Hay mucha gente en las salas y ves que hay líneas rojas para no acercarse a las obras].
62. You are lining up with your friends to enter the bar. There are a lot of other people also waiting to get in.
[Esperas con tus amigos en la fila de un bar. Hay muchas personas que también están esperando para entrar].
66. You are walking in the town square when you see someone setting up a table to play the "shell game".
[Estás caminando por la plaza y de repente observas que se está instalando un puesto para jugar "dónde quedó la bolita"].
67. You are in a subway car and you realize that to get to your destination you have two stations left.
[Vas en un vagón del metro y te das cuenta de que para llegar a tu destino te faltan dos estaciones].
71. In the library, where you should be quiet, everyone is reading or working on their computers.
[En la biblioteca, donde se debe guardar silencio, todas las personas están leyendo o trabajando en su computadora].
78. You are driving home and there is a lot of traffic. You feel annoyed, just like the other drivers.
[Regresas a casa en auto y hay mucho tráfico. Te sientes muy fastidiado igual que los demás conductores].

4-noCD RESPONSES - PERCENTAGES (%)												
STIMULI	CHEATING DETECTION		ADVERSELY AFFECTED					BENEFICIARY				
	YES	NO	CH	SE	YOU	UND	NOB	CH	SE	YOU	UND	NOB
**8**	5.9	94.1	0.4	5.2	4.8	8.4	81.3	3.6	9.2	8.4	20.5	58.2
**13**	1.6	98.4	0.0	6.3	1.6	4.7	87.5	0.8	0.8	12.9	5.5	80.1
**15**	2.3	97.7	0.4	0.8	9.3	2.3	87.2	1.2	3.1	12.8	7.0	76.0
**19**	11.8	88.2	1.2	7.4	1.2	5.4	84.8	5.8	2.7	3.1	8.9	79.4
**23**	3.5	96.5	0.0	1.2	4.0	3.6	91.3	1.6	4.3	9.0	6.3	78.8
**26**	3.2	96.8	0.4	0.4	0.4	5.3	93.5	1.2	2.5	22.5	9.0	64.8
**27**	6.7	93.3	0.8	2.7	2.7	7.0	86.7	2.7	3.1	10.2	12.9	71.1
**29**	2.7	97.3	0.4	0.8	0.0	3.9	94.9	1.2	4.0	4.7	15.0	75.1
**33**	3.1	96.9	0.8	0.4	9.9	4.0	85.0	1.2	0.0	0.0	5.1	93.7
**42**	6.3	93.8	0.4	2.8	6.7	4.7	85.4	2.4	1.2	3.2	8.4	84.9
**50**	4.1	95.9	1.2	13.9	2.9	11.1	70.9	4.1	14.4	0.4	11.5	69.5
**54**	9.5	90.5	1.2	4.9	3.3	10.7	79.9	7.0	5.7	0.4	11.9	75.0
**59**	6.0	94.0	0.4	1.2	1.2	4.1	93.1	1.2	4.0	0.8	10.1	83.8
**62**	6.5	93.5	0.4	1.6	2.5	3.7	91.8	1.6	0.4	1.2	6.9	89.8
**66**	13.7	86.3	0.0	9.3	0.4	9.7	80.6	9.7	6.0	0.0	11.3	73.0
**67**	4.5	95.5	0.0	0.4	7.7	4.5	87.4	0.4	0.0	5.7	5.7	88.3
**71**	5.2	94.8	0.4	2.8	0.4	2.4	94.0	2.4	3.2	3.6	12.9	77.8
**78**	4.9	95.1	0.0	0.8	18.3	8.3	72.5	0.8	1.2	1.7	8.3	88.0
CH = Cheater; SE = Someone Else; YOU = First Person/Yourself; UND = Undefined; NOB = Nobody												

**4-noCD** ELICITED EMOTIONAL REACTIONS				
STIMULI	GLAD	INDIFFERENT	ANNOYED	ANGRY
**8**	25.1	62.8	9.3	2.8
**13**	20.3	77.7	1.6	0.4
**15**	29.1	57.1	10.6	3.1
**19**	13.9	79.0	6.3	0.8
**23**	28.6	66.3	3.6	1.6
**26**	52.7	46.1	1.2	0.0
**27**	28.1	66.4	4.0	1.6
**29**	27.0	71.4	0.4	1.2
**33**	9.9	80.2	5.1	4.7
**42**	8.4	71.2	17.2	3.2
**50**	4.6	83.8	6.7	5.0
**54**	8.7	78.4	9.1	3.7
**59**	22.0	73.4	3.3	1.2
**62**	10.0	76.7	10.4	2.9
**66**	5.2	82.7	6.0	6.0
**67**	14.0	78.6	5.3	2.1
**71**	36.9	60.7	1.6	0.8
**78**	3.8	52.5	32.4	11.3

5-CDbF. Situations that include cheating, but result in the first person's benefit, meaning that the interviewed participant is the *beneficiary*.

4. In the library, where it should be silent, the person you fancy is next to you and finally decides to speak to you.
[En la biblioteca, donde se debe guardar silencio, junto a ti está la persona que te gusta y por fin decide hablarte].
7. A group of people don't follow the rule of showering before swimming. This allows you to shower directly when you get out of the pool.
[Un grupo no obedece la regla de ducharse antes de nadar, ello te permite utilizar rápidamente la regadera cuando sales de nadar].
11. You are driving home. Lots of cars take a prohibited left turn, allowing you to beat the traffic.
[Regresas a casa en auto. Muchos autos se dan una vuelta prohibida y ello permite que avances y superes el tráfico].
18. You are in a busy subway car. Someone pushes you, causing you to end up next to someone you like, who smiles at you.
[Vas en el metro. Alguien te empuja, eso provoca que quedes junto a alguien que te gusta y te sonría].
25. In the vehicle testing center, someone pays a bribe for their car to pass the test. But the supervisor gets confused and puts your car first.
[En el verificentro, alguien da mordida para pasar la verificación, pero el gerente se confunde y te pasa primero a ti].
34. You are in an exam and your tutor writes some answers on the board because he is in a good mood. They are exactly the ones you didn't know.
[Estás en un examen, tu profesor anota algunas respuestas en el pizarrón porque está de buenas, justo las que no te sabías].
43. At a party, a child sneakily charges guests to use the bathroom. When he's found out, they decide he should give you all the money.
[En una fiesta, un niño cobra indebidamente por usar el baño. Cuando lo descubren deciden que te entregue el dinero recolectado].
46. You are driving home. Someone jumps the red light and leaves you in the best position to turn.
[Regresas a casa en auto. Una persona se pasa el alto y te deja en la mejor posición para dar vuelta].
56. You are the substitute goalkeeper in your team. They discover that the first-team goalkeeper does doping, so you are promoted to first-team goalkeeper.
[Eres el portero suplente de tu equipo. Descubren que el portero titular se dopa. Por ello te ascienden a portero titular].

58. You are a candidate. They discover that another candidate was buying votes and as a result, people start voting for you.
[Eres un candidato. Descubren que otro candidato estaba comprando votos. Como consecuencia, la gente comienza a votar por ti].
68. Taking photos with flash is not allowed in the museum. A famous photographer takes a photo using his flash and he gives it to you.
[En el museo está prohibido tomar fotos con flash. Un fotógrafo famoso toma una foto con flash para regalártela].
69. On the metrobus, a non-disabled person takes the disabled seat. As a result of the argument, a seat become free which you then take.
[En el Metrobús, un no-discapacitado ocupa el lugar de discapacitados. Como consecuencia de la discusión, se libera un lugar que tú tomas].
74. In the store, the checkout person is not handing over the receipts. But he gives you extra change and winks at you.
[En la tienda, la cajera no está entregando tickets. Pero a ti te da cambio de más y te guiña el ojo].
77. In the museum, an alarm sounds and people go to see what happened. This lets you better enjoy your favorite painting.
[En el museo suena una alarma, la gente va a ver qué paso y ello te permite disfrutar mejor tu obra favorita].
80. You are queueing with your friends to enter the bar. One of them is friends with the bouncer and he lets your group in.
[Esperas con tus amigos en la fila de un bar. Uno de ellos conoce al cadenero y deja pasar a tu grupo].
81. Someone invites you to bet on the “shell game” in the town square. Although they cheat, you win the bet.
[En la plaza, un sujeto te invita a apostar para adivinar "dónde quedó la bolita". Aunque hace trampa, tú ganas la apuesta].
85. In the subway station, some people start jumping the turnstile. The guard opens the gate and lets you in without paying.
[En el metro, algunas personas empiezan a saltarse el torniquete, el policía abre la puerta y te deja pasar sin pagar].
88. A non-disabled driver parks in a disabled space and there is a traffic jam. You have to back up and suddenly find a space.
[Un no-discapacitado ocupa el lugar de discapacitados, se da un embotellamiento. Te echas en reversa y encuentras un lugar].

5-CDbF RESPONSES - PERCENTAGES (%)												
STIMULI	CHEATING DETECTION		ADVERSELY AFFECTED					BENEFICIARY				
	YES	NO	CH	SE	YOU	UND	NOB	CH	SE	YOU	UND	NOB
**4**	50.6	49.4	3.1	25.1	23.1	11.0	37.6	7.8	4.3	62.0	8.6	17.3
**7**	64.2	35.8	5.3	29.8	10.6	26.1	28.2	25.6	7.3	28.0	13.4	25.6
**11**	84.9	15.1	6.7	38.2	6.3	22.4	26.4	15.4	2.4	73.5	5.5	3.2
**18**	17.2	82.8	0.8	7.8	9.4	3.1	78.8	3.1	0.8	77.7	3.5	14.8
**25**	89.4	10.6	49.2	37.1	2.8	6.5	4.4	4.8	1.6	90.3	1.2	2.0
**34**	61.3	38.7	0.4	7.9	14.6	9.4	67.7	2.8	3.5	79.9	3.5	10.2
**43**	71.8	28.2	55.0	26.3	4.8	6.0	8.0	12.7	16.7	51.2	9.9	9.5
**46**	79.1	20.9	6.5	16.6	4.5	17.8	54.7	8.1	1.2	80.5	2.8	7.3
**56**	54.1	45.9	60.7	17.6	1.2	4.9	15.6	5.3	2.9	82.0	3.7	6.1
**58**	78.8	21.2	73.4	13.3	3.2	4.8	5.2	5.3	2.4	86.6	2.4	3.2
**68**	93.3	6.7	2.8	51.8	4.3	23.3	17.8	9.8	2.0	82.4	3.1	2.7
**69**	78.8	21.2	4.4	63.8	6.1	13.1	12.7	16.7	10.5	53.9	8.8	10.1
**74**	90.1	9.9	5.9	58.9	2.4	19.4	13.4	4.0	1.6	85.7	4.0	4.8
**77**	23.5	76.5	2.1	15.7	2.5	14.5	65.3	2.1	2.5	73.4	5.0	17.0
**80**	92.3	7.7	1.2	65.6	4.0	18.8	10.4	7.2	2.8	82.0	3.6	4.4
**81**	86.6	13.4	71.0	14.1	4.8	3.6	6.5	5.6	0.4	87.9	2.4	3.6
**85**	86.8	13.2	5.2	38.1	0.4	27.8	28.6	4.8	2.0	83.2	6.4	3.6
**88**	68.3	31.7	3.9	43.5	5.7	17.4	29.6	28.1	3.1	43.0	8.8	17.1
CH = Cheater; SE = Someone Else; YOU = First Person/Yourself; UND = Undefined; NOB = Nobody												

**5-CDbF** ELICITED EMOTIONAL REACTIONS				
STIMULI	GLAD	INDIFFERENT	ANNOYED	ANGRY
**4**	63.3	18.4	14.5	3.9
**7**	9.3	61.0	25.6	4.1
**11**	31.0	42.9	18.3	7.9
**18**	83.5	11.4	3.1	2.0
**25**	51.2	33.3	12.6	2.8
**34**	73.8	17.1	7.1	2.0
**43**	42.0	32.4	22.0	3.6
**46**	33.7	49.6	11.8	4.9
**56**	63.5	22.1	10.7	3.7
**58**	70.4	16.6	9.3	3.6
**68**	42.9	33.5	22.0	1.6
**69**	16.4	48.7	25.2	9.7
**74**	36.9	31.7	24.5	6.8
**77**	63.1	29.9	4.6	2.5
**80**	51.4	33.9	9.8	4.9
**81**	72.8	19.9	3.7	3.7
**85**	46.6	39.7	12.1	1.6
**88**	19.3	50.9	21.5	8.3

2. Procedure for the pilot studies

A summary table of the 3 Pilot Studies, stage 4, is the following **Table A**:

Table A. Number of participants, total duration of trial, ages, educational level of participants, number of stimuli, advances and caveats of the instrument to detect cheating

	Number of participants	***Total duration of the trial***	***Age (in years)***	***Educational level***	***Number of stimuli presented***	***Time of presentation of each stimulus***	***Advances and caveats***
First pilot	4	1.40 hours	32-46	Graduate students	105	8 seconds	At first, the stimuli consisted on a very heterogeneous word count (even 5 lines) and plenty of time was spent on reading them. Some stimuli were rewritten.
Second pilot	6	1.25 hours	24-77	Graduate students	105	8 seconds	Then, the stimuli became shorter in order to control the stimuli extension (word count). It was difficult for the participants to get accustomed to the computer and test format, and some examples at the beginning of the test were provided. For the next pilot study, the number of stimuli was reduced to 90 because three situations were suppressed as subjects did not detect cheating in them.
Third pilot	6	40 minutes	28-41	4 graduate and 2 undergraduate students	90	7 seconds	The number of words and characters for each stimulus was set similar. The 105 stimuli were reduced to 90. It was necessary to introduce an initial description of the instrument.
